# Abnormal *N*‐glycan fucosylation, galactosylation, and sialylation of IgG in adults with classical galactosemia, influence of dietary galactose intake

**DOI:** 10.1002/jmd2.12237

**Published:** 2021-07-22

**Authors:** Eileen P. Treacy, Sebastian Vencken, Annet M. Bosch, Matthias Gautschi, Estela Rubio‐Gozalbo, Charlotte Dawson, Darragh Nerney, Hugh Owen Colhoun, Loai Shakerdi, Gregory M. Pastores, Roisin O'Flaherty, Radka Saldova

**Affiliations:** ^1^ National Centre for Inherited Metabolic Disorders, The Mater Misericordiae University Hospital Dublin Ireland; ^2^ Department of Paediatrics Trinity College Dublin Dublin Ireland; ^3^ UCD School of Medicine University College Dublin Dublin Ireland; ^4^ Department of Medicine Trinity College Dublin Dublin Ireland; ^5^ Department of Pediatrics, Division of Metabolic Disorders Emma Children's Hospital, Amsterdam Gastroenterology, Endocrinology & Metabolism, Amsterdam UMC, University of Amsterdam Amsterdam The Netherlands; ^6^ Department of Paediatrics and Institute of Clinical Chemistry Inselspital, University Hospital Bern Bern Switzerland; ^7^ Department of Pediatrics/Laboratory of Clinical Genetics Maastricht University Medical Centre Maastricht The Netherlands; ^8^ Department of Endocrinology University Hospitals Birmingham NHS Foundation Trust Birmingham UK; ^9^ NIBRT GlycoScience Group, National Institute for Bioprocessing, Research and Training Dublin Ireland; ^10^ Department of Chemistry Maynooth University Kildare Ireland; ^11^ UCD School of Medicine, College of Health and Agricultural Sciences (CHAS), University College Dublin (UCD) Dublin Ireland

**Keywords:** biomarkers, classical galactosemia, dietary galactose, immunoglobulin G, *N‐*glycosylation

## Abstract

**Background:**

Classical galactosemia (CG) (OMIM #230400) is a rare disorder of carbohydrate metabolism, due to deficiency of galactose‐1‐phosphate uridyltransferase (EC 2.7.7.12). The pathophysiology of the long‐term complications, mainly cognitive, neurological, and female infertility remains poorly understood.

**Objectives:**

This study investigated (a) the association between specific IgG *N*‐glycosylation biomarkers (glycan peaks and grouped traits) and CG patients (n = 95) identified from the GalNet Network, using hydrophilic interaction ultraperformance liquid chromatography and (b) a further analysis of a *GALT* c.563A‐G/p.Gln188Arg homozygous cohort (n = 49) with correlation with glycan features with patient Full Scale Intelligence Quotient (FSIQ), and (c) with galactose intake.

**Results:**

A very significant decrease in galactosylation and sialylation and an increase in core fucosylation was noted in CG patients vs controls (*P* < .005). Bisected glycans were decreased in the severe *GALT* c.563A‐G/p.Gln188Arg homozygous cohort (n = 49) (*P* < .05). Logistic regression models incorporating IgG glycan traits distinguished CG patients from controls. Incremental dietary galactose intake correlated positively with FSIQ for the p.Gln188Arg homozygous CG cohort (*P* < .005) for a dietary galactose intake of 500 to 1000 mg/d. Significant improvements in profiles with increased galactose intake were noted for monosialylated, monogalactosylated, and monoantennary glycans.

**Conclusion:**

These results suggest that *N*‐glycosylation abnormalities persist in CG patients on dietary galactose restriction which may be modifiable to a degree by dietary galactose intake.

**Abbreviations**
CG: classical galactosemia
FSIQ: Full Scale Intelligence Quotient
Gal: UDP‐galactose
Gal‐1‐P: galactose‐1‐phosphate
GalNAc: *N*‐acetylgalactosamine
GalNet: International Galactosemia Network
GALT: galactose‐1‐phosphate uridyltransferase
Glc: UDP‐glucose
GlcNAc: *N*‐acetylglucosamine
HILIC‐UPLC: hydrophilic interaction ultraperformance liquid chromatography
RBC: red blood cell

**Synopsis**

Significant decreases in galactosylation and sialylation and increases in core fucosylation and a decrease in bisected IgG glycans (involving the *GALT* c.563A‐G/p.Gln188Arg homozygous cohort) were observed in CG patients. Incremental dietary galactose intake correlated positively with FSIQ and with improvements in monosialylated, monogalactosylated, and monoantennary glycan structures.

## 1 INTRODUCTION

Classical galactosemia (CG) (OMIM 230400) is a rare disorder of carbohydrate metabolism caused by galactose‐1‐phosphate uridyltransferase (GALT) deficiency (EC 2.7.7.12).^1^ Deficiency of GALT results in an accumulation of intermediates of the galactose metabolism (Leloir) pathway, such as galactose‐1‐phosphate (Gal‐1‐P), galactitol and galactonate.^1^ The only available current treatment option is a long‐term galactose restricted diet. Dietary intervention can be lifesaving in the neonate. However, long‐term complications persist in treated adult patients to include significant cognitive impairment, movement disorders, decreased bone mineral density, and infertility in females. These complications are present regardless of genotype or age at the onset of treatment.^1^, ^2^, ^3^, ^4^, ^5^, ^6^ The accumulation of toxic galactose intermediates coupled with deficiency of UDP‐hexose sugars is proposed to contribute to the development of these complications with possible disruption of glycosylation central to the post‐translational modification of protein and lipids.^7^, ^8^ The current tests of measuring red blood cell (RBC) Gal‐1‐P and urinary galactitol levels, apart from predicting gross deviations from diet and monitoring initial decreases of RBC Gal‐1‐P in the neonate, do not reveal milder deviations or correlate with clinical outcome.^9^, ^10^, ^11^, ^12^

Selected studies have identified *N*‐glycan assembly and processing defects using the study of transferrin in CG.^13^, ^14^, ^15^ Four adults with CG on a galactose‐restricted diet showed deviations from the control reference range for specific plasma *N*‐ and *O*‐glycans identified by MALDI‐TOF and quantified by HPLC‐MS/MS.^16^

Immunoglobulin G (IgG) plays an important role in the human immune system and modifiable *N*‐glycans attached to the Fc region can switch functionality of IgG between pro‐ and anti‐inflammatory statuses. The absence of sialic acid changes the physiological role of IgG from anti‐inflammatory to pro‐inflammatory^17^ and changes in IgG galactosylation also have significant implications.^18^

We previously identified *N*‐glycan assembly defects in neonates using serum IgG and ongoing significant *N*‐glycan processing defects in treated young children and adults with galactosemia.^19^, ^20^, ^21^, ^22^, ^23^ We identified a significant increase in core fucosylated neutral glycans and a significant decrease in core fucosylated and afucosylated bisected glycans in IgG glycans from galactosemia adult Irish and Dutch CG patients^23^, with subsequent clinical validation of an automated high‐throughput IgG hydrophilic interaction ultraperformance liquid chromatography (HILIC‐UPLC) method.^24^

We also reported the significant dysregulation of a number of related relevant *N*‐glycan biosynthesis genes in peripheral blood mononuclear cells in CG patients including the genes *ALG9*, *MGAT1*, and *MGAT3*.^23^, ^25^

Applying the circulating IgG *N*‐glycan markers to a deep phenotyping study to include IQ as a measure of intelligence, neurological examination assessing motor development, tremor, and speech abnormalities of 56 Dutch CG patients (children and adults), statistically significant differences were noted in specific *N*‐glycan peaks between patients and controls. However, specific individual glycan peaks were not found to correlate directly with neurological outcomes.^26^

The above studies have indicated that the glycosylation abnormalities in treated galactosemia patients may be subtle and individual within a background of individual genetic variation of glycosylation pathways. Also overlaps between control ranges for patients and controls in the context of individual glycosylation variation, effects of milder *GALT* gene variants and epigenetic effects on glycosylation may confound direct group comparisons for glycosylation abnormalities between galactosemia patients as a group in comparison to controls.

There is ongoing controversy regarding the optimum amount of galactose required in the diet for CG patients, in particular for adults.^6^, ^9^, ^11^, ^27^, ^28^, ^29^, ^30^, ^31^, ^32^, ^33^, ^34^ In the recently reported International Galactosemia Network (GalNet) Registry outcome study of 509 CG patients, it was noted that patients following a strict galactose‐restricted diet (lactose restricted and restrictions in fruit and vegetables) developed neurological complications more frequently (*P* < .001; odds ratio [OR] 2.81 [1.64‐4.50]) than patients with a less strict diet (no restrictions of fruit and vegetables).^6^

In this current study, we sought to identify if the validated IgG *N*‐glycan assay could be simplified by the grouping of glycan features (e.g., fucosylation, sialylation, measurement of agalactosylated, mono or digalactosylated glycan peaks), and if any of these group features could act as monitoring biomarkers to determine optimum personalized glycosylation profiles with differing dietary galactose intake in CG patients, using an extended galactosemia population from the GalNet Network. To potentially correct for the confounding effect of variation of the *GALT* genotype, we also studied a subcohort of patients who are homozygous for the “severe” CG *GALT* mutation, c.563A‐G, and p.Gln188Arg. As a secondary analysis, we also sought to determine if there was any association with significant *N*‐glycan grouped features with measured total Full Scale Intelligence Quotient (FSIQ) in the *GALT* c563A‐G, p.Gln188Arg homozygous cohort.

## 2 MATERIALS AND METHODS

### 2.1 Patient characteristics

Inclusion criteria: A total of 95 CG patients originating from five centers in four countries included in the GalNet Network were included in this study (see Table 1A for demographic characteristics). All Dutch, Irish, and UK patients had CG phenotypes with two pathogenic *GALT* gene mutations and/or erythrocyte GALT enzyme activity below the limit of quantitation of the enzyme assay (<3.3%; <1.1 μmol/h.gHb). A number of the Swiss subjects had GALT residual activity (less than 10% of normal) (see Table 1A).

**TABLE 1A:** Study participants and demographics

	*GALT* genotype	No	Gender		Age at testing: mean (range) years
			F	M	
Controls	N/A	81	42	39	29 (18‐40)
Irish		25	5	20	35
Scottish		56	37	19	27
Patients		**95**	53	42	26 (16‐63)
Expert centre/country					
Dublin, Ireland^a^	p.Gln188Arg/p.Gln188Arg	**31**	15	18	22 (16‐36)
	p.Gln188Arg/p.Arg333Trp	2			
Amsterdam, the Netherlands^b^	p.Gln188Arg/p.Gln188Arg	**10**	12	8	26 (16‐47)
	p.Gln188Arg/p.Lys285Asn	3			
	p.Arg205Ter/Trp316Ter	1			
	p.Gln188Arg/p.Leu195Pro	2			
	p.Gln188Arg/p.Lys127Glu	1			
	p.Gln188Arg/p.Ser135Trp	2			
	N/A	1			
Maastricht, the Netherlands^a^	p.Gln188Arg/p.188Arg	**4**	7	0	20 (16‐26)
	p.Leu195Pro/p.Lys285Asn	3			
Bern, Switzerland	p.Gln188Arg/p.Gln188Arg	**5**	13	9	32 (16‐59)
	p.Gln188Arg/p.Lys285Asn	3			
	p.Gln188Arg /p.Leu195Pro	2			
	p.Gln188Arg/p.Ala320Thr	3			
	p.Lys285Asn/p.Lys285Asn	1			
	p.Met142Lys/p.Ala320Thr	1			
	p.Lys285Asn/p.His319Gln	2			
	p.Ala320The/p.His319Gln	1			
	p.Gln188Arg/p.Met142Lys	1			
	p.Arg258Cys/p.Leu195Pro	2			
	p.Gln188Arg/p.Leu264Val	1			
Birmingham, UK	N/A	13	6	7	31 (19‐63)

^a^ For this cohort (n = 33 subjects from Dublin NCIMD and 7 from Maastricht), this cohort is similar to as reported in Stockmann et al, 2015^22^ and Maratha et al, 2016^23^ with the exclusion of cases age under age 16 at the time of sampling.

^b^ This cohort is as described in Welsink et al, 2020.^26^

**TABLE 1B:** Full Scale Intelligence Quotient (FSIQ) scores and daily intake of galactose for the genotype p.Gln188Arg/pGln188Arg

	No	Mean	Range	%
p.Gln188Arg/pGln188Arg	49			
Nationality				
Irish	31			
Dutch	13			
Swiss	5			
FSIQ	49	78	47‐126	
Daily intake of galactose				
<200 mg	29			59.2
200‐500 mg	10			20.4
501‐1000 mg	10			20.4

The most recent FSIQ assessment as noted by the study clinicians assessed using standardized psychological testing was documented for each patient. The standardized tests used at the four centers were the Wechsler Intelligence Scale for Children (WISC) and the Wechsler Adult Intelligence Scale (WAIS) according to the age at testing. These tests included the subdomain tests: Verbal Comprehension, Perceptual Reasoning, Working Memory as well as the FSIQ. FSIQ is a measure of the individual's overall cognitive ability based on the individual's performance on all the subtests.

The dietary galactose daily intake as recorded by the treating clinician was based on the analysis of a detailed food record, analyzed by Dietplan6 with the analysis of free galactose values for fruit, vegetables, legumes, and other possible galactose sources at three of the four sites.^11^

For the Amsterdam site, the subjects maintained a lactose‐free diet with no restrictions in fruit and vegetables, with expected galactose daily intake of less than 100 mg/d.^9^

The control samples (n = 81) were obtained from a pool of healthy adult volunteers, 56 from a Scottish Orkney Island healthy population epidemiological study and 25 from a healthy Irish population health insurance screening panel (see Table 1A).

### 2.2 IgG N‐glycan analysis

**FIGURE 1:**
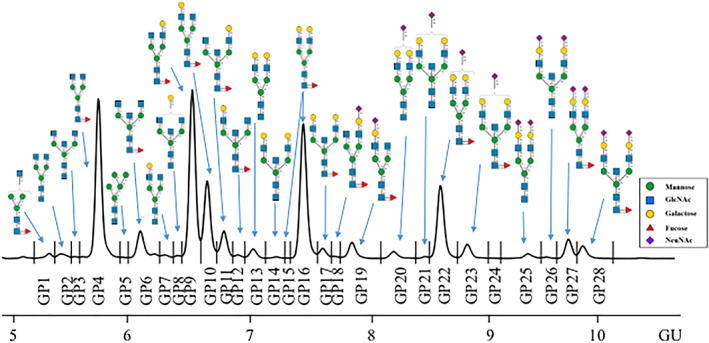
Representative hydrophilic interaction ultraperformance liquid chromatography (HILIC‐UPLC) chromatogram of released undigested human immunoglobulin G (IgG) *N*‐glycome from classical galactosemia (CG) patients. The main glycan structures in each glycan peak (GP1‐28) are pictured in the box insert

As previously reported, the IgG analysis was carried out from prepared serum samples using Protein G plates on a STARlet Microlab robotic liquid handling platform linked to UPLC.^22^, ^24^ Twenty‐eight *N*‐glycan peaks (GP), previously characterized by UPLC and mass spectrometry were identified (Figure 1).^35^

The glycan peaks represent the relative percentage areas derived from the HILIC‐UPLC profiles. The data are compositional and convey the relative amounts of glycan structures in a sample rather than the absolute quantities.^36^ The glycan peak groups were grouped according to common features of interest, as branching (monoantennary [MA, GP1], biantennary [BA, GP2‐4+GP6‐23+GP25‐28] complex glycans, or oligomannosylated [OM, GP5/2] glycans), galactosylation (agalactosylated [G0, GP1‐6], monogalactosylated [G1, GP7‐12+GP18‐19], and digalactosylated [G2, GP13‐17+GP20‐23+GP25‐28] structures), sialylation (asialylated [S0, GP1‐17], monosialylated [S1, GP18‐23] glycans), fucosylation (core fucosylated [CF, GP1+GP4+GP6+GP9‐12+GP15‐19+GP22‐23+GP27‐28] glycans), and bisected glycans (B, GP3+GP6+GP8+GP11‐12+GP14+GP17+GP21+GP23+GP26+GP28). GP5/2 denotes that the oligomannosylated glycans represent approximately half of the GP5 peak. Other specific features were also investigated: core fucosylated neutral glycans (Fn, GP1+GP4+GP5+GP9‐10+GP16), core fucosylated bisected neutral glycans (FBn, GP6+GP11‐12+GP17), and afucosylated bisected neutral glycans (Bn, GP3+GP8+GP14).

### 2.3 Statistical analysis

SPSS version 25 (SPSS Inc, Chicago, Illinois) and R 4.0.0 (R Core Team, 2020) were used to perform all statistical analyses. Medians and ranges were presented. A multivariate analysis test was used to assess differences in values (GP peaks and groups) between cases and controls followed by the use of the Tukey post hoc test.

To explore any association between individual *N*‐glycan peaks and FSIQ, a linear regression approach was used. Given the relatively large number of covariates (*N*‐glycan peaks) compared to the sample size, variable selection was initially applied using linear LASSO regression with cross‐validated mean square error minimization to select covariates that best explain the continuous response variable, FSIQ. *N*‐glycan peaks were standardized to avoid scaling issues and included as continuous independent variables along with patient daily galactose intake treated as a categorical variable with three levels of intake: <200, 200‐500, and 501‐1000 mg galactose per day. Subsequent to variable selection, the association between FSIQ and selected covariates was analyzed using ordinary least squares regression. Proportion of variance explained (*R* ^2^) and individual effect estimates, along with their confidence intervals, were reported.

To assess the associations between galactosemia and relevant features in the case‐control dataset, the individual *N*‐glycan peaks associated with each feature were included in logistic regression models, one for each feature, which had the case‐control grouping factor as response variable. *N*‐glycan peaks were standardized to avoid scaling issues.

The cross‐validated C‐statistic and McFadden's pseudo‐*R* ^2^ were reported for each model. To assess generalization error caused by model overfitting, the C‐statistics and their standard deviations were calculated using 10‐fold 5X‐repeated cross validation.

We then analyzed whether galactose intake caused a difference in the grouped features for the p.Gln188Arg homozygous group (n = 49). The Shapiro‐Wilk test of normality was initially conducted to assess normality of group distributions. A one‐way ANOVA test was used for parametric data; the Kruskal‐Wallis test was used for nonparametric data. Bonferroni correction was used to control for the type 1 error rate due to multiple comparisons.

## 3 RESULTS

The patient cohort for this study is described in Table 1A. The most common *GALT* genotype is homozygosity for the p.Gln188Arg *GALT* gene pathogenic variant (n = 50). The FSIQs as most recently recorded and available for subjects (n = 49) was recorded. Of the 49 most recent assessments that were available, 71% included the adult WAIS assessment (age of testing 16‐36), and 29% included the WISC assessment (age of testing 8‐15). According to the International Standard Classification of Education (ISCED) scale as used in the GalNet Registry^6^ only 14 of 47 individuals with available data (30% of total) achieved a level of education higher than ISCED 3 (upper secondary education).

The approximate daily galactose intake as available and as reported by the treating centers was grouped for 49 of the p.Gln188Arg homozygotes as follows: Group 1 (n = 29): <200 mg; Group 2 (n = 10): 200‐500 mg; Group 3 (n = 10): 501‐1000 mg.

The serum IgG *N*‐glycome from all patients was released using a high‐throughput method and resulting chromatograms were separated into 28 peaks (Figure 1).^22^, ^24^ Glycan features such as branching, fucosylation, galactosylation, and sialylation were also determined.

Comparing CG/p.Gln188Arg/p.Gln188Arg homozygotes with controls, several peaks and features were significantly altered, namely glycan peaks GP4 and GP26 were increased and GP1, 2, 3, 5, 8, 12, 18, 19, 20, 22, 24, and 25 were decreased (Table 2A). In the derived (grouped) features, core fucose (CF), biantennary (BA), agalactosylated (G0), neutral (S0), and core fucosylated neutral glycans (Fn) were increased and oligomannose (OM), monoantennary (MA), mono and digalactosylated (G1, G2), monosialylated (S1), and bisected (B) glycans were decreased (Table 2B). All G0/G1, G0/G2, and G0/G1/G2 ratios were increased in CG patients (Table 2B).

**TABLE 2:** *N*‐glycan peaks of significance in CG patient groups

A. All glycan peaks					
	CG patients (n = 95)	p.Gln188Arg/p.Gln188Arg (n = 49)	Controls (n = 81)	*P*‐values (CG vs controls/p.Gln188Arg/p.Gln188Arg vs controls)	Main glycans
GP1	0.49 (0.16‐1.49)	0.51 (0.16‐1.05)	0.66 (0.26‐1.95)	<.0005	FA1
GP2	0.70 (0.36‐1.26)	0.76 (0.42‐1.26)	0.79 (0.45‐1.01)	<.0005/.087	A2
GP3	0.31 (0.05‐1.61)	0.39 (0.09‐1.25)	0.39 (0.22‐0.57)	<.05/.683	A2B
GP4	21.30 (12.54‐33.81)	21.64 (12.54‐33.11)	18.80 (8.60‐36.15)	<.0005/<.005	FA2
GP5	0.13 (0.01‐0.23)	0.13 (0.02‐0.21)	0.19 (0.01‐0.26)	<.0005	M5
GP8	0.26 (0.01‐0.52)	0.23 (0.01‐0.52)	0.27 (0.01‐22.20)	<.05	A2BG1
GP12	0.54 (0.26‐1.22)	0.49 (0.29‐0.82)	0.53 (0.34‐1.04)	.789/<.005	FA2[3]BG1
GP18	0.26 (0.22‐2.75)	0.34 (0.09‐2.56)	0.41 (0.22‐2.75)	<.0005	FA2[6]G1S1
GP19	2.14 (0.51‐3.54)	1.98 (0.81‐2.90)	2.40 (0.51‐3.54)	<.0005	FA2[3]G1S1
GP20	0.75 (0.13‐2.54)	0.79 (0.24‐1.28)	0.86 (0.13‐2.54)	<.005/.062	A2G2S1
GP22	7.52 (1.49‐12.41)	7.79 (3.53‐12.31)	8.76 (1.49‐12.41)	<.05/.151	FA2G2S1
GP24	0.04 (0.01‐0.23)	0.04 (0.01‐0.23)	0.06 (0.03‐0.36)	<.0005/<.005	n.d.
GP25	0.49 (0.13‐1.34)	0.53 (0.14‐1.28)	0.56 (0.06‐1.29)	<.05/.452	A2G2S2
GP26	0.14 (0.04‐0.33)	0.12 (0.04‐0.33)	0.11 (0.04‐0.70)	<.05/.618	A2BG2S2

B. Derived features from all glycan peaks measured				
Features	CG patients (n = 95)	p.Gln188Arg/p.Gln188Arg (n = 49)	Controls (n = 81)	*P*‐values (CG vs controls/p.Gln188Arg/p.Gln188Arg vs controls)
CF	95.30 (91.80‐97.21)	95.41 (91.81‐97.06)	95.03 (63.41‐97.15)	<.005/<.05
OM	0.07 (0.01‐0.12)	0.06 (0.01‐0.010)	0.10 (0.01‐0.13)	<.0005
MA	0.49 (0.16‐1.49)	0.51 (0.16‐1.05)	0.66 (0.26‐1.95)	<.0005
BA	99.34 (98.30‐99.72)	99.34 (97.95‐100.08)	99.09 (97.81‐99.71)	<.0005
G0	27.00 (17.28‐45.97)	27.46 (18.32‐39.73)	25.50 (13.53‐46.03)	<.005
G1	39.66 (32.57‐44.31)	39.47 (32.57‐42.80)	40.11 (32.78‐43.15)	.163/<.05
G2	32.77 (18.46‐46.91)	32.92 (19.35‐44.73)	34.56 (21.17‐49.80)	<.05/.054
S0	84.59 (71.85‐92.34)	84.37 (74.08‐91.34)	82.41 (74.49‐88.60)	<.0005/<.005
S1	12.43 (6.59‐20.26)	12.45 (7.21‐18.61)	14.69 (9.53‐19.18)	<.0005
S2	2.90 (1.06‐7.78)	2.90 (1.07‐7.15)	2.84 (1.67‐6.20)	NS
B	15.16 (9.48‐18.66)	14.51 (9.48‐18.66)	15.20 (10.92‐38.71)	.194/<.05
Fn	70.26 (60.63‐78.34)	70.32 (55.86‐78.34)	67.82 (29.72‐77.10)	<.0005/<.005
Fbn	10.84 (6.26‐19.81)	10.34 (6.26‐15.89)	11.07 (2.88‐16.53)	NS
Bn	0.69 (0.33‐1.90)	0.76 (0.33‐1.69)	0.85 (0.44‐22.50)	NS
G0/G1	0.69 (0.48‐1.29)	0.70 (0.50‐1.08)	0.65 (0.37‐1.40)	<.0005
G0/G2	0.82 (0.37‐2.49)	0.82 (0.41‐2.05)	0.73 (0.27‐2.17)	<.005/<.05
G0/G1/G2	0.020 (0.010‐0.070)	0.021 (0.011‐0.050)	0.018 (0.007‐0.066)	<.005

*Notes*: Main glycans were assigned as described in Pucic et al.^35^*N*‐glycan features were calculated as described in Materials and Methods. Data reported in median and ranges. GPs with gray highlighted areas are increased and GPs without highlighted areas are decreased in CG patients. Only one *P*‐value is listed if both comparisons display the same significance.‐

Abbreviations: All *N*‐glycans have two core GlcNAcs; F at the start of the abbreviation indicates a core fucose α1,6‐linked to the inner GlcNAc; Mx, number (x) of mannose on core GlcNAcs; Ax, number of antenna (GlcNAc) on trimannosyl core; B, bisected GlcNAc linked β1,4 to β1,3 mannose; Gx, number (x) of β1,4 linked galactose on antenna; Sx, number (x) of sialic acids linked to galactose. All sialic acids are linked α2‐6 to galactose. Glycan terminology abbreviations used: Sialylation: S0 (neutral glycans), S1 (monosialylated), S2 (disialylated), Galactosylation: G0 (agalactosylated), G1 (monogalactosylated), G2 (digalactosylated). Branching: MA, monoantennary; BA, biantennary. OM, oligomannose. Fucosylation: CF, core‐fucose. Bisecting glycans: B, total bisecting glycans. Other specific features: Bn, afucosylated bisected neutral glycans; FBn, core fucosylated bisected neutral glycans; Fn, core fucosylated neutral glycans.

When the overall CG cohort (all genotypes) were compared to the p.Gln188Arg/p.Gln188Arg cohort, the significant changes in the glycomes were similar, which mostly reached significance in the whole cohort, possibly due to higher numbers, but the same trend was observed also in the p.Gln188Arg/p.Gln188Arg cohort even if not always statistically significant (Table 2A and 2B). The exceptions were GP12, monogalactosylated glycans (G1), and total bisected glycans (B), which were significantly decreased only in the p.Gln188Arg/p.Gln188Arg cohort (Table 2A and  2B).

To further explore the association between the glycan features and CG, a series of logistic regression models were used as described in the Methods section. This was to quantify the combined ability of the individual peaks of each glycan feature to classify a p.Gln188Arg *GALT* homozygous galactosemia patient from a healthy control.

The results indicate that the S0 and BA features have the strongest association with CG as determined by their cross validated c‐statistics and pseudo‐*R* ^2^s, both of which indicate a strong association (Table 3). Larger cohorts may show a stronger distinction in associative performance between these models.

**TABLE 3:** Tenfold cross‐validated C‐statistics and McFadden's pseudo‐*R* ^2^ for differentiation of p.Gln188Arg *GALT* homozygotes from controls using glycan features

Feature	C‐statistic	C‐statistic SD	Pseudo *R* ^2^	Pseudo *R* ^2^ SD
S0	0.917	0.082	0.47	0.24
BA	0.879	0.079	0.33	0.21
G1	0.870	0.066	0.27	0.15
CF	0.869	0.063	0.27	0.12
Fn	0.868	0.109	0.43	0.20
G0	0.844	0.101	0.23	0.16
G2	0.838	0.089	0.18	0.11
S1	0.831	0.092	0.21	0.18
OM	0.806	0.108	0.23	0.20
B	0.735	0.109	0.12	0.08
MA	0.724	0.152	0.12	0.17
S2	0.658	0.142	0.03	0.04
Bn	0.508	0.157	0.03	0.03
FBn	0.504	0.138	0.03	0.05

For the p.Gln188Arg/p.Gln188Arg homozygotes as a group for analysis of correlation with FSIQ, a positive correlation was observed for galactose intake and for branched glycans (*R* = 0.397, *P* = .006 for galactose intake and *R* = 0.35 for branched glycans). The result for branched glycans was deemed to be too low to warrant further analysis.

Galactose intake was subsequently included as a covariate in a linear regression model with FSIQ as the response variable (Table 4). When the estimated mean increase in FSIQ for 200‐500 and 501‐1000 mg galactose intake was compared to <200 mg galactose intake (*P* < .005), only the 501‐1000 mg galactose intake group showed a statistically significantly higher FSIQ compared to the <200 mg galactose intake group (Table 4). However, the contemporary galactose intake could only explain 23% of the FSIQ variability between patients (*R* ^2^: 0.23).

**TABLE 4:** Association of galactose intake with IQ in p.Gln188Arg/p.Gln188Arg cohort

Galactose intake (<200 mg gal is the baseline)	Estimated mean change IQ	95% CI	*P*‐value (uncorrected)
200‐500 mg	1.56	−12.17 to 15.30	.820
501‐1000 mg	22.99	9.78‐36.19	.001

**FIGURE 2:**
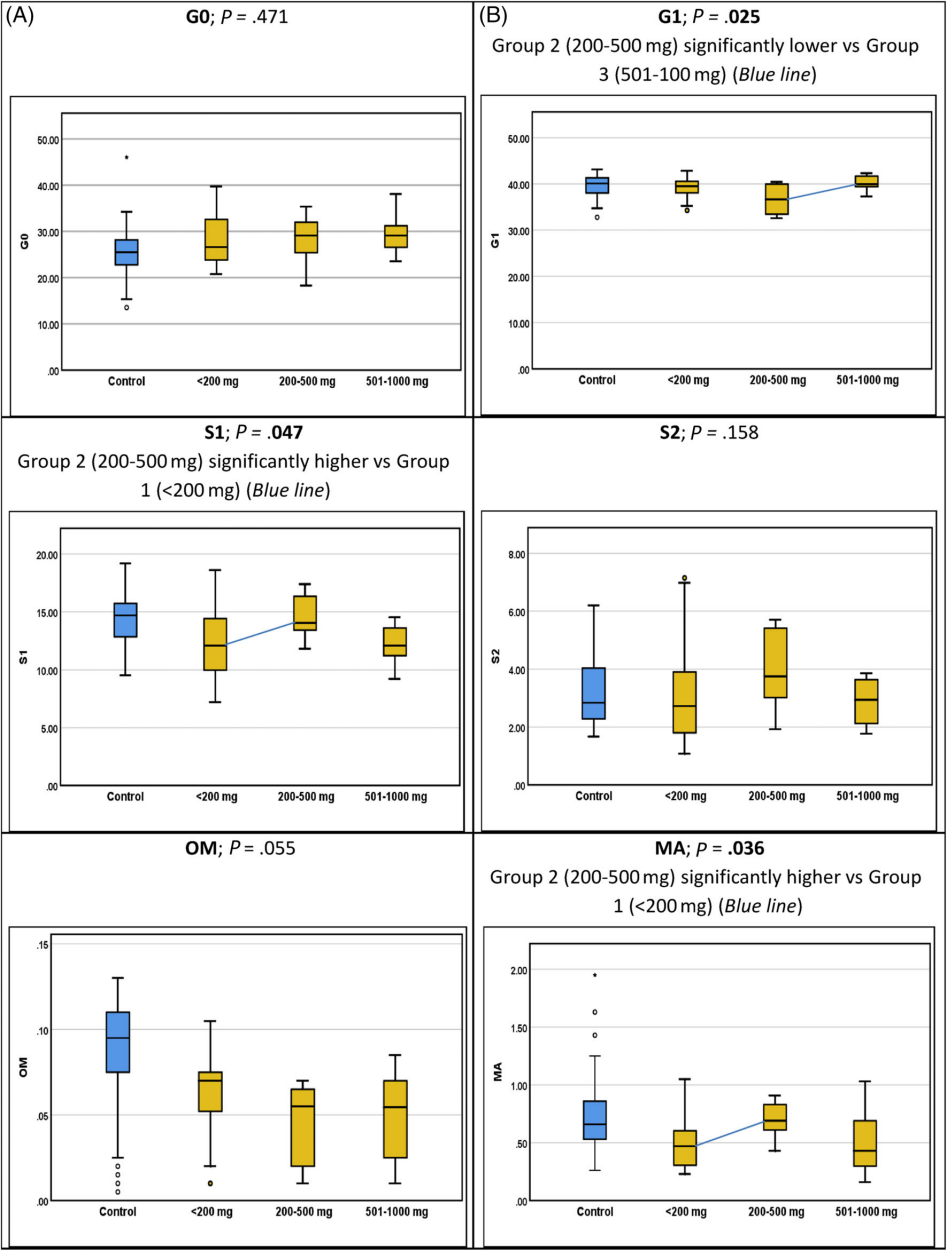
Boxplots of glycan features for controls and patients with galactose intakes of <200, 200‐500, and 501‐1000 mg. The boxplots display the median and ranges. A, Controls are shown in blue; patients are shown in yellow. B, Significant difference in values according to galactose intake are shown (*P* < .05)

The analysis of differences between galactose intake in glycan peaks and groups for the three groups of p.Gln188Arg/p.Gln188Arg homozygotes (n = 49) with differing galactose intake was assessed (Figure 2; Table 5). The trend for the majority of GPs was to approach the control range for both degrees of galactose liberalization. All resultant values were in the control ranges for the GPs (see Table 2A). However, statistical significance differences were only reached for the G1, S1, and MA groups. For the G1 feature, for group 2 (galactose intake of 200‐500 mg day), this group had significantly lower scores (36.66 [32.57‐40.42]) vs group 3 (intake 501‐1000 mg/d) (39.91 [37.28‐42.34]), *P* ≤ .05. For the S1 feature, group 2 had significantly higher scores (14.05 [11.83‐17.39]) vs group 1 (12.08 [7.23‐18.61]) (*P* ≤ .05), approaching the median of the control range. For the MA feature, group 2 had a significantly higher level 0.69 (0.43‐0.91) than group 1 (*P* < .05) with values approaching the median of the control range in group 2.

**TABLE 5:** Association of IgG glycome (grouped features) with galactose intake in p.Gln188Arg/p.Gln188Arg cohort

Galactose daily intake	<200 mg (n = 29)	200‐500 mg (n = 10)	501‐1000 mg (n = 10)	*P*‐value
CF	95.14 [91.81‐97.06]	95.34 [93.12‐96.23]	96.00 [93.56‐96.97]	.156
OM	0.07 [0.01‐0.10]	0.06 [0.01‐0.07]	0.06 [0.01‐0.085]	.055
MA	0.47 [0.23‐1.05]	0.69 [0.43‐0.91]	0.43 [0.16‐1.03]	.036*
BA	99.35 [99.1‐100.08]	99.13 [98.74‐99.72]	99.48 [97.92‐99.60]	.175
G0	26.58 [20.78‐39.73]	29.12 [18.32‐35.36]	29.12 [23.54‐38.06]	.471
G1	39.57 [34.28‐41.66]	36.66 [32.57‐40.42]	39.91 [37.28‐42.34]	.025*
G2	33.51 [19.35‐42.61]	32.19 [24.51‐42.53]	31.41 [21.93‐37.1]	.526
S0	85.26 [74.08‐91.34]	81.72 [77.33‐86.22]	84.63 [81.65‐89.01]	.088
S1	12.08 [7.23‐18.61]	14.05 [11.83‐17.39]	12.09 [9.22‐14.52]	.047*
S2	2.72 [1.07‐7.15]	3.76 [1.92‐5.7]	2.94 [1.77‐3.86]	.158
B	15.27 [10.27‐18.66]	13.64 [11.23‐15.29]	13.16 [9.48‐17.82]	.090
Fn	69.45 [55.86‐78.34]	69.04 [63.56‐74.27]	72.53 [68.62‐77.12]	.082
FBn	10.87 [6.27‐15.89]	9.84 [8.22‐11.95]	9.76 [6.26‐12.67]	.092
Bn	0.79 [0.38‐1.69]	0.72 [0.41‐0.96]	0.72 [0.33‐1.56]	.203
G0/G1	0.67 [0.55‐0.99]	0.82 [0.50‐1.08]	0.72 [0.60‐0.95]	.305
G0/G2	0.79 [0.49‐2.05]	0.84 [0.41‐1.23]	0.94 [0.63‐1.74]	.466
G0/G1/G2	0.02 [0.01‐0.05]	0.02 [0.01‐0.03]	0.03 [0.02‐0.04]	.546

* Significant at *P* ≤ 0.05 level. Data are presented as medians and ranges.

The previously reported G0/G1, G1/G2, and G0/G1 and G2 ratios were not informative in discriminating galactose tolerance.

## 4 DISCUSSION

CG is considered to be a secondary glycosylation disorder. The effects of galactose restriction in the intoxicated neonate are well documented. Also there is evidence that there are differences in how patients of identical *GALT* genotype manifest *N*‐glycan profiles with increased galactose intake.^20^, ^31^

This study aimed to identify if grouped glycan (complex carbohydrate) IgG features in adult CG patients ascertained from the GalNet Consortium could serve as predictive clinical biomarkers for galactose tolerance and also as a secondary analysis to look at IQ (FSIQ) as an outcome with IgG features for the Q188R homozygous group.

In the current study, we have replicated our previous findings showing a decrease in galactosylation in CG as demonstrated by significantly increased ratios of G0/G1, G0/G2, and G0/G1/G2 (Table 2B) consistent with the report by Stockmann et al.^22^ We also report significant increases in the glycans GP4 and 26 and decreases in GP1, 5, 18, and 24 (Table 2A); consistent with findings by Maratha et al and Welsink et al, respectively.^23^, ^26^ In this cohort, we also report additional significant peaks (GP2, 4, 8, 12, 19, 20, 22, and 25). There are also other glycan peaks which were previously found to be significant in our previous studies, but were not shown to be significant in this cohort (GP7, 11, 15, and 21; Table 2A).

The glycome of immunoglobulins is noted to be highly variable with high heritability^37^ with polymorphisms of the glycan genes encoding the glycosyltransferases ST6GAL1, B4GALT1, FUT8, and MGAT3, noted to represent the most important loci associated with variation in IgG traits.^38^ Thus, differences in background glycosylation pathways may account for individual variation in glycan peaks as shown by the wide ranges of values of controls in Table 2A.

In this study, we did not note significant differences in gender or age in the study and control groups. We considered it to be more practical/informative thus to analyze differences in grouped glycan features (Table 2B) rather than the individual glycan peaks.

To decrease confounders of possible residual enzymatic activity for some patients in the total cohort, our final analysis with FSIQ and dietary tolerance was based on homozygotes only for p.Gln188Arg, as CG patients with this genotype are well described in the literature as having a severe CG phenotype. While studying the p.Gln188Arg homozygous cohort may eliminate some of these confounders, we have however also noted significant contemporary differences in glycosylation in siblings homozygous for p.Gln188Arg emphasizing the potential significance of epigenetic effects on glycosylation and alternate accessory glycosylation pathways.^19^, ^20^

For the groups feature analysis, the total bisected glycans (B) were found to be decreased in the p.Gln188Arg/p.Gln188Arg cohort (Table 2B) consistently with decrease in the *MGAT3* gene expression which we previously reported.^23^ It is considered that the bisecting *N*‐acetylglucosamine (GlcNAc) structure represents a specific type of *N*‐glycosylation modification involved in biological processes including cell adhesion, fertilization, neurite outgrowth, and tumorigenesis.^18^

The observed increase in core fucosylation is consistent with our previous findings, namely the increases in Fn and total fucosylation (CF) (Table 2B).

When the total study cohort was compared to the p.Gln188Arg cohort, the significant changes in the glycomes were consistent, mostly reaching significance in the whole cohort (Table 2A), possibly due to higher numbers. However, the same trend was observed also in the p.Gln188Arg/p.Gln188Arg cohort; though with smaller numbers not always reaching significance.

Although we also noted significant findings of ongoing abnormal branching, fucosylation, and galactosylation of IgG *N*‐glycans among treated CG patients, we only found a direct correlation between branching glycans and galactose intake with the measured IQ of these patients, with only galactose intake being statistically significant. This finding is not unexpected. While there are statistically significant differences between specific glycan peaks and grouped features between treated CG and controls, the measured outcome (FSIQ) is likely influenced by prenatal galactose exposure or intoxication, neonatal galactose intoxication, and possible ongoing abnormalities of systemic *N*‐glycan processing abnormalities and cell signaling abnormalities.

In this study, we found a decrease in sialylated glycans in the CG patients, with the most significant correlation existing between the non sialylated (S0) glycans and the phenotype of CG.

We consider that the role of sialic acid and galactose in glycan processing as central determinants of the outcome measured (IQ) has a strong biological plausibility.^39^, ^40^, ^41^

Many of the linear and branched glycans on cell surface glycoproteins and glycolipids of vertebrates are terminated with sialic acids, nine‐carbon sugars with a carboxylic acid, a glycerol side‐chain, and an *N*‐acyl group that provide for varied molecular interactions. Sialic acid is found in large quantities in human milk oligosaccharides as sialylated‐glycoconjugates and is an essential component of brain gangliosides and sialylated glycoproteins, particularly as precursors for the synthesis of the polysialic acid glycans that posttranslationally modify the cell membrane associated neural cell adhesion molecules). In addition, gangliosides, sialylated glycosphingolipids, are the most abundant sialoglycans of nerve cells. The multiple antennae of classical “complex type” *N*‐linked glycans are often terminated with “NeuAc α2–3 (or α2‐6) Gal β1‐4 GlcNAc” sequences. The most abundant *O*‐glycans are bound to proteins via an *N*‐acetylgalactosamine (GalNAc)‐Ser/Thr linkage.^40^

Abnormalities of sialic acid biosynthesis are embryonically lethal in mice, and are associated with a variety of human diseases.^42^, ^43^ The four predominant gangliosides in the brain share the same neutral glycan core (Gal β1‐3 GalNAc β1‐4 Gal β1‐4 Glc β1‐1 Cer) with varying numbers of sialic acids attached to the internal and terminal galactose residues. Genome‐wide linkage analysis implicates sialylation as a determinant of higher cognitive functions.

In particular, the ST3GAL3 enzyme transfers sialic acids to terminal Gal residues in β1‐3 or β1‐4 linkage to GlcNAc or in β1‐3 linkage to GalNAc. This is a characteristic of *O*‐ and *N*‐linked glycoproteins and gangliosides.^44^ We have previously demonstrated dysregulation of this gene and other sialyltransferases in CG patients.^45^

The long‐term outcomes in treated patients with CG have indicated a high incidence of lower intellectual outcome, and more recently a high percentage of motor symptoms including dystonia and tremor in children and adults.^2^, ^3^, ^4^, ^5^, ^6^, ^26^, ^46^ In parallel with this, a number of studies have indicated gray and white matter changes in MRI scans of the brain in individuals with CG.^26^, ^47^, ^48^ It is currently unclear if the white and gray matter changes observed in patients with CG are progressive. Deficiency of glycolipids containing galactose or GalNAc has been demonstrated in a postmortem brain examination of a patient with galactosemia.^49^ In addition to galactose, sialic acid is essential for glycolipid synthesis. Fucosylated, galactosylated, and sialylated complex *N*‐glycans have been identified as significant constituents of the human brain glycome.^49^, ^50^

For the grouped features (listed in Table 2B), although the G ratios previously reported significantly differentiate CG patients from controls, these ratios did not significantly differentiate patients with differing galactose intake (Table 5). The features which were significantly different between the subgroups of patients with differing galactose dietary intake were the features S1 (*P* = .047), G1 (*P* = .025), and MA (*P* = .036). In conjunction with the informative results in Tables 2B and 4, it may be feasible to utilize the features S0, S1, G0, and G1 as biomarkers of galactose tolerance, individualized to each patient as their own control.

For the combined data set, the study sample in the two smaller groups (n = 10) limit the conclusions in this regard for the group in general, and as stated earlier there is an overlap between controls and cases for the reference range. Although this study involving multiple partners of the GalNet Network is the largest study of CG (n = 95 patients including 49 Q188R homozygotes), a larger controlled study would be required to study this further.

## 5 STUDY LIMITATIONS

As stated above, as a rare disease, the study number is still limited with patients studied with possible differing genetic backgrounds. Thus, a larger study is required to test the clinical utility of the proposed biomarkers to examine galactose tolerance in these individuals. Larger cohorts may show a stronger distinction in associative performance between the models used. For this study, the analysis of galactose dietary intake was based on dietary analysis documentation available at the time of blood sampling. Accurate retrospective quantitation of galactose intake remains problematic.^51^ Also the analysis presented likely represents the galactose intake prior to the sampling. It cannot provide insight into early life or life‐long galactose intake which may directly affect neurological outcomes.

## 6 CONCLUSIONS

**FIGURE 3:**
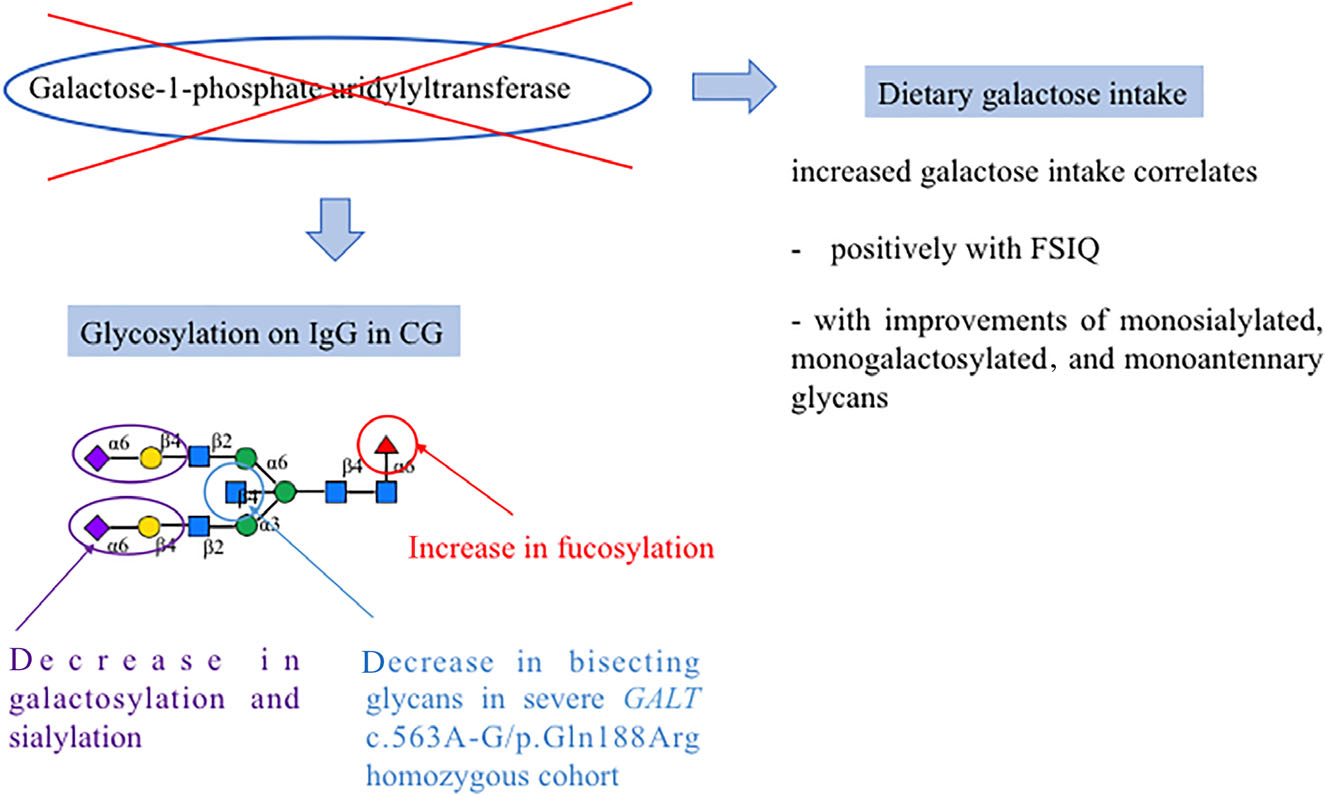
Summary of main findings (immunoglobulin G [IgG] features) and correlations with Full Scale Intelligence Quotient (FSIQ)

In this study, we have described characteristic features in galactosemia patients, namely an increase in core fucosylation and a decrease in galactosylation of IgG as well as a decrease in bisected glycans in the *GALT* gene p.Gln188Arg homozygous cohort. Figure 3 summarizes these results, galactose intake correlation with FSIQ, with improvements of monosialylated, monogalactosylated, and monoantennary glycans.

We propose that galactosylation and sialylation of glycans of major physiological relevance may be modified by moderate exogenous galactose dietary intake. These studies provide further insight from a rare inborn error of metabolism into the central role of galactosylation in glycan synthesis. The influence of these changes on corresponding protein/cell function needs to be further delineated so that management of affected individuals can be tailored accordingly.

## CONFLICT OF INTEREST

The authors declared no potential conflict of interest. This study does not involve animals.

## ETHICS STATEMENT

Ethical approval for this study was obtained from the ethics committee of the Mater Misericordiae University Hospital, Dublin, Ireland (Reference 1/378/1811). The patients gave their full informed consent to partake before they were enrolled in the study.

## Data Availability

The original dataset for the IgG analysis is available, on request.
